# Creatine supplementation post-exercise does not enhance training-induced adaptations in middle to older aged males

**DOI:** 10.1007/s00421-014-2866-1

**Published:** 2014-03-16

**Authors:** Matthew B. Cooke, Brian Brabham, Thomas W. Buford, Brian D. Shelmadine, Matthew McPheeters, Geoffrey M. Hudson, Christos Stathis, Mike Greenwood, Richard Kreider, Darryn S. Willoughby

**Affiliations:** 1 College of Health and Biomedicine, Victoria University, PO Box 14428, Melbourne, 8001 Australia; 2 Department of Exercise and Sport Science, University of Mary Hardin-Baylor, Temple, TX USA; 3 Department of Aging and Geriatric Research, University of Florida, Gainesville, FL USA; 4 Department of Health, Human Performance and Recreation, Baylor University, Waco, TX USA; 5 School of Public Health and Health Services, The George Washington University, Washington, DC USA; 6 Department of Health and Kinesiology, University of Texas A&M, College Station, TX USA

**Keywords:** Supplementation, Aging, Muscle, Hypertrophy, Creatine, Supplementation

## Abstract

**Purpose:**

The present study evaluated the effects of creatine monohydrate (CrM) consumption post-exercise on body composition and muscle strength in middle to older males following a 12-week resistance training program.

**Methods:**

In a double-blind, randomized trial, 20 males aged between 55 and 70 years were randomly assigned to consume either CrM-carbohydrate (CHO) [20 g days^−1^ CrM + 5 g days^−1^ CHO × 7 days, then 0.1 g kg^−1^ CrM + 5 g CHO on training days (average dosage of ~8.8 g)] or placebo CHO (20 g days^−1^ CHO × 7 days, then 5 g CHO on training days) while participating in a high intensity resistance training program [3 sets × 10 repetitions at 75 % of 1 repetition maximum (1RM)], 3 days weeks^−1^ for 12 weeks. Following the initial 7-day “loading” phase, participants were instructed to ingest their supplement within 60 min post-exercise. Body composition and muscle strength measurements, blood collection and vastus lateralis muscle biopsy were completed at 0, 4, 8 and 12 weeks of the supplement and resistance training program.

**Results:**

A significant time effect was observed for 1RM bench press (*p* = 0.016), leg press (*p* = 0.012), body mass (*p* = 0.03), fat-free mass (*p* = 0.005) and total myofibrillar protein (*p* = 0.005). A trend for larger muscle fiber cross-sectional area in the type II fibers compared to type I fibers was observed following the 12-week resistance training (*p* = 0.08). No supplement interaction effects were observed.

**Conclusion:**

Post-exercise ingestion of creatine monohydrate does not provide greater enhancement of body composition and muscle strength compared to resistance training alone in middle to older males.

## Introduction

Aging is associated with progressive loss of neuromuscular function that often leads to progressive disability and loss of independence. Sarcopenia is now a commonly used term to describe the loss of skeletal muscle mass and strength that occurs in concert with biological aging (Janssen et al. 2002; Bales and Ritchie 2002; Borst 2004). By the seventh and eighth decade of life, muscle strength can be reduced, on average, by approximately 20–40 % for both men and women (Doherty 2003). Although age-associated decreases in strength per unit of muscle mass (i.e. muscle quality) may play a role, a large proportion of strength loss can be accounted for by decreased muscle mass (Doherty 2003). Multiple factors have been implicated in the development of sarcopenia and the associated impact on function. Loss of skeletal muscle fibers secondary to decreased numbers of motor neurons appears to be a major contributing influence, but other factors, including decreased physical activity, altered hormonal status, decreased total caloric and protein intake, inflammatory mediators, and factors leading to altered protein synthesis must also be considered (Buford et al. 2010).

In the past decade, strength training has been investigated extensively as a means of attenuating sarcopenia (Hunter et al. 2004; Candow and Chilibeck 2007). Indeed, high intensity resistance training is known to increase myofibrillar protein synthesis as well as muscle hypertrophy (Candow and Chilibeck 2007). However, literature suggests that despite training, muscle loss may still occur in older adults, indicating factors including nutrition may also play an important role in combating sarcopenia (Campbell and Leidy 2007).

One potentially efficacious method of leveraging nutrition to enhance the efficacy of resistance training is supplementation with creatine monohydrate (CrM). In athletic populations, CrM has been extensively studied over the past 20 years, with its effects on high intensity, short-term exercise well-documented (Rawson and Volek 2003; Williams and Branch 1998; Chrusch et al. 2001). To date, available evidence regarding the ergogenic effect of CrM supplementation in older adults is equivocal as some studies have reported improvement (Rawson and Wehnert 1999; Gotshalk et al. 2007; Brose and Parise 2003) or no change (Elliot et al. 2008; Bemben et al. 2010; Olsen et al. 2006) in various measures of body composition and muscle performance. In recent years, it has been suggested that the timing of nutritional supplementation in close proximity of an exercise bout maybe more important than absolute daily intake of the supplements (Cribb and Hayes 2006; Candow and Chilibeck 2008; Antonio and Ciccone 2013). Numerous studies have shown the benefit of protein timing on adaptations to resistance exercise in both young and older populations (Stark et al. 2012). However, research into the ergogenic potential of creatine ingestion in close proximity to exercise completion is limited. In young males and females, creatine ingestion (0.2 g kg^−1^, average dosage of ~15.5 g) following a single limb exercise for 6 weeks increased lean muscle mass (males only) and elbow flexor muscle thickness to a greater extent than placebo ingestion (Chilibeck et al. 2004). Conversely, creatine taken before and after exercise training at a lower dosage (0.1 g kg^−1^, average dosage of ~5.7 g) failed to provide any further increases in body mass, lean muscle mass and total muscle thickness when compared to placebo ingestion in older males (Candow et al. 2008). Recently, Antonio and Ciccone (2013) showed that consuming as little as 5 g of creatine post-exercise may affect the adaptive response to exercise in young male bodybuilders. However, the purpose of this study was to compare pre- vs. post-exercise consumption of creatine and thus a true control (i.e., no supplement) was not included (Antonio and Ciccone 2013). Subsequently, the effectiveness of creatine ingestion in close proximity of resistance exercise at a lower dosage is unclear.

While continuous CrM supplementation (acute or long term) appears to enhance resistance training-induced muscle adaptations in young and older individuals, the benefit of timing creatine ingestion relative to the resistance exercise session is unclear. Therefore, the primary purpose of this study was to determine the effect of post-exercise CrM consumption on body composition and muscle strength in middle to older males. We hypothesized that post-exercise CrM consumption concurrent with a resistance training program would result in greater training enhancement in upper and lower body muscle strength, lean muscle mass, muscle fiber cross-sectional area (CSA) when compared to CHO ingestion.

## Materials and methods

### Participants

Twenty-eight apparently healthy male participants between the ages of 55–70 years began this study. A total of eight participants were unable to complete the study for the following reasons: time commitment (4), uncomfortable with muscle biopsy procedure (2), and non-study related injury (2). A total of 20 participants (10 per group) completed all testing sessions and all 20 participants were used for subsequent analysis of all variables. Sample size was calculated using G Power software. Based on expected lean muscle mass changes (Chrusch et al. 2001) for the creatine and placebo groups of 55.85 ± 1.65 and 51.45 ± 1.35 kg, respectively, the calculated effect size is 2.92. With an *α* of 0.05, a sample size of 8 per group yields a power of 0.92.

Participants were recreationally active (i.e. performed exercise 1–2 days weeks^−1^) but non-resistance trained (i.e. no regular resistance training for at least 1 year) Exclusionary criteria included smoking during previous year, use of nutritional supplements or supplemental androgens within the previous 6 months, current use of statins, recent history of cancer (within 2 years), neurological disease or cardiac arrhythmia interfering with physical function, peripheral vascular disease, congestive heart failure, complicated diabetes, chronic inflammatory disease, recent stroke (within 1 year), renal/kidney disease, and any absolute contraindications to exercise according to American College of Sports Medicine (ACSM) guidelines.

All eligible participants were asked to provide oral and written informed consent based on documents approved by the Institutional Review Board of Baylor University. All experimental procedures involved in the study conformed to the ethical consideration of the Helsinki Code. Verbal explanation of the purpose of the research, the protocol to be followed, and the experimental procedures to be used were given to the participants.

### Entry and familiarization session

Participants expressing interest in participating in this study were interviewed on the phone and/or via email to determine whether they appeared to qualify to participate in this study. Participants believed to meet eligibility criteria were then invited to attend an entry/familiarization session. Participants were asked to bring signed physician approval of participation to the familiarization session. During this session, the potential participants signed informed consent statements and completed personal and medical history forms. Participants meeting entry criteria were familiarized to the study protocol, and the tests to be performed, via a verbal and written explanation outlining the study design. Participants were instructed to refrain from exercise for 48 h and fast for 8 h prior to testing.

### Experimental design

Upon reporting to the lab for the exercise testing session, body mass, heart rate and blood pressure were determined prior to analyzing body composition using dual X-ray absorptiometry (DEXA) (Hologic, Waltham, MA). Following the DEXA scan, venous blood and skeletal muscle biopsy samples were obtained by standard/sterile procedures. Participants then performed upper body and lower body 1 repetition maximum (1RM) strength measurements. Participants were then matched on lower body 1RM strength, age and fat-free mass and randomly assigned to consume either CrM-CHO or CHO placebo while participating in a high intensity resistance training program (3 sets × 10 repetitions at 75 % of 1RM), 3 days weeks^−1^ for 12 weeks. At 4 (T2), 8 (T3) and 12 (T4) weeks, participants returned to the lab to perform the same battery of tests as performed at baseline (T1) 48 h after the last workout. A total of four blood and muscle biopsy samples were taken during the study.

### Muscle strength assessment

At 0, 4, 8 and 12 weeks participants performed 1RM muscular strength tests using an isotonic 45° leg press (Nebula Fitness, Inc., Versailles, OH) and bench press (Nebula Fitness, Inc., Versailles, OH). Participants began the 1RM leg press and bench press test by first completing five to ten repetitions at approximately 50 % of their previously established 1RM. Following a 2-min rest, three to five repetitions were performed at approximately 70 % of their 1RM. From this time forward, the weight was increased gradually, until an official 1RM was reached. The rest period between each successful lift was 2 min. Test–retest reliability for performing these strength assessments on participants within our laboratory has demonstrated low mean coefficients of variation and high reliability for the bench press (1.9 %, intraclass *r* = 0.94) and leg press (0.7 %, intraclass *r* = 0.91), respectively.

### Resistance training program

Participants completed a partially supervised resistance training program. Partially supervised means that each participant was supervised for the first 2 weeks to ensure correct technique was being implemented. Participants were then monitored (i.e. correct technique used, correct weight lifted etc.) at the gym at 6, 8 and 11 weeks. Participants utilized either local fitness centers of which they were a member or facilities on the Baylor University campus. Participants participated in a partially supervised resistance training program three times per week for the duration of the study. Participants completed resistance exercises similar to those utilized by Chrusch et al. (2001). Participants completed the bench press, lat pull-down, biceps curl, triceps press down, leg press, leg extension and leg curl. Three sets of 10 repetitions were completed for each exercise. An intensity of 75 % of 1RM was utilized during training. Upon successful completion of 3 sets of 10 repetitions, an increase of 5 % of weight was utilized to ensure progressive overload during the study took place. A rest period of 1 min separated each set. Participants were asked to keep training logs detailing amount of weight utilized, number of repetitions completed and number of sets completed.

### Nutritional supplementation

Participants were randomly assigned to receive, in a double-blind manner, either 20 g of CrM (Alschem, Degussa Inc., Atlanta, GA) combined with 5 g of glucose (DGC AST Sport Science, Golden, CO) for 7 days followed by 0.1 g kg^−1^ (average dosage of ~8.8 g) of CrM with 5 g of glucose on training days or 20 g of glucose only for 7 days followed by 5 g of glucose on training days. Each supplement dosage was provided in small identical plastic bags. Supplement was given to participants in a 4-week block period and thus dosage could be adjusted following body-weight analysis. Each supplement was similar in color and texture. A small shaker bottle was provided to participants with instructions on how to mix the supplement in about 500 ml of water. Participants receiving CrM completed a 7-day loading period with 20 g of CrM being ingested daily in 4 × 5 g increments (i.e. 9 am, 12, 3 and 6 pm). Participants in both the experimental group (CrM-CHO) and the placebo control group (CHO) began the designed supplementation protocol immediately after baseline testing which took place on Day 0. Following the 7 day loading phase, participants were instructed to ingest either the supplement or placebo within 60 min following the resistance exercise session for the duration of the 12-week study period. Justification of the supplementation protocol is threefold: First, a lower dosage of 0.07–0.1 g kg^−1^ (approximately 5–8 g days^−1^) results in minor adverse events (i.e., loose stools, muscle cramping, and muscle pulls/strains) (Chrusch et al. 2001; Candow et al. 2008). Second, a dosage as little as 5 g days^−1^, albeit continuous, has shown to be effective at increasing lean muscle mass and strength in older males (Brose and Parise 2003; Chrusch et al. 2001). And finally, a single 5 g oral dose of CrM in healthy adults results in a peak plasma creatine level of approximately 120 mg/l at 1–2 h post-ingestion (i.e., during that post-exercise window) (Jager et al. 2007). Compliance monitoring was accomplished by having participants return empty containers of the supplement or placebo at each testing session. Compliance to the supplementation protocol was monitored by supplement logs and verbal confirmation. Compliance was also verified through the administration and collection of questionnaires from each participant regarding any noticeable side-effects.

### Tissue and blood sampling

Skeletal muscle biopsies were collected accordingly to previously published procedures (Cooke et al. 2011). Briefly, percutaneous muscle biopsies were obtained using the Bergstroem technique under local anesthesia of 1 % Xylocaine from middle portion of the vastus lateralis muscle of the dominant limb at the midpoint between the patella and the greater trochanter of the femur at a depth between 2 and 3 cm. Muscle samples were obtained by standard/sterile procedures by personnel who were experienced in performing the procedure. After removal, the tissue samples were immediately frozen in liquid nitrogen and then stored at −80 °C for future analyses.

Participants were required to fast overnight prior to donating approximately 20 ml of venous blood from the antecubital vein using standard phlebotomy procedures. Blood was collected into serum separation tubes and centrifuged at 1,100*g* for 15 min. Serum was separated and stored at −80 °C in polypropylene tubes for later analysis.

### Serum insulin-like growth factor 1 and testosterone

Serum samples were analyzed in duplicate for free/bioactive IGF-1 (Diagnostic Systems Laboratories, Webster, TX), and free testosterone (Alpha Diagnostic International, San Antonio, TX) using an ELISA (enzyme linked immunoabsorbent assay). For IGF-1, assay sensitivity is 0.06 ng/ml, and does not cross-react with albumins or GH (growth hormone) binding proteins. For testosterone, sensitivity is 0.17 pg/ml. For IGF-1 and testosterone the subsequent absorbances, which were directly proportional to the concentration of analyte in the sample, were measured at a wavelength of 450 nm using a microplate reader (Wallac Victor 1420, Perkin Elmer, Boston MA). A set of standards of known concentrations for IGF-1 and testosterone were utilized to construct standard curves by plotting the net absorbance values of the standards against their respective protein concentrations. By applying a four-part parameter curve using MikroWin microplate data reduction software (Microtek Lab Systems, Germany), the free IGF-1 and free testosterone concentrations in the serum samples were calculated. The overall intra-assay percent coefficient of variation was 4.9 and 3.7 % for IGF-1 and testosterone, respectively.

### Fiber type and cross-sectional area

Muscle tissue used for determination of muscle fiber type and CSA were taken at 0 and 12 weeks. Tissue obtained from the biopsy procedure was mounted using OCT medium and snap frozen in isopentane, pre-cooled in liquid nitrogen, and stored at −80 °C for histochemical analysis to classify muscle fiber types into fast (II) and slow (I) on the basis of the stability of their ATPase activity, as previously described (Cribb and Hayes 2006). Fiber type CSA was determined from sections containing a mean of 210 (range 130–400) fibers. Samples were measured on two separate occasions for day-to-day reproducibility. ICC (intraclass correlation coefficient) and SD (standard deviation) for fiber type distribution were type I *r* = 0.81, SD 1.6 % and type II *r* = 0.93, SD 1.4 %.

### Myofibrillar protein content

Total cellular RNA (ribonucleic acid) was extracted from biopsy samples with a monophasic solution of phenol and guanidine isothiocyanate contained within the TRI-reagent (Sigma Chemical Co., St. Louis, MO), and then isolated with 100 % isopropanol. The interphase was removed and total (soluble + insoluble) muscle protein was then isolated from the organic phase with 100 % isopropanol and washed with a 0.3 M guanidine HCl/95 % ethanol solution. Myofibrillar (soluble) protein was further isolated with repeated incubations in 0.1 % SDS at 50 °C and separated by centrifugation. Total myofibrillar protein content was determined spectrophotometrically based on the Bradford method at a wavelength of 595 nm (Bradford 1976). A standard curve was generated (*R* = 0.98, *p* = 0.001) using bovine serum albumin (Bio-Rad, Hercules, CA), and total myofibrillar protein content was expressed relative to muscle wet-weight.

### Dietary records

Participants were required to keep dietary records for 7 days prior to the exercise testing sessions. The 7-day dietary records were evaluated with the ESHA Food Processor 8.6 (ESHA Research, Salem, OR) dietary assessment software program to determine the average daily macronutrient consumption of fat, carbohydrate and protein, and consumption of antioxidant-related micronutrients vitamin A, vitamin C, vitamin E and selenium in the diet.

### Statistical analysis

Data was analyzed by utilizing separate 2 × 4 [group (CrM-CHO, CHO)] × test (week 0, week 4, week 8, week 12) mixed design factorial multivariate analysis of variance (MANOVA). MANOVAs were analyzed for this study based on dependent variables that are likely to be related to one another. In addition, the use of a MANOVA analysis prevents the potential for the increasing of type I error rate that might result with the use of repeated univariate procedures. ANOVA on each dependent variable was conducted as a follow-up test to any significant MANOVA. To control for alpha inflation of the ANOVA, the Bonferroni test was utilized as a follow-up test. Post-hoc tests of any interaction effects demonstrated in the ANOVA were investigated via an independent samples *t* test. In addition to reporting probability values, an index of effect size was reported to reflect the magnitude of the observed effect. The index of effect size utilized was the partial Eta squared (*η* ^2^), which estimates the proportion of variance in the dependent variable that can be explained by the independent variable. Partial Eta squared effect sizes were determined to be: weak = 0.17, medium = 0.24, strong = 0.51, very strong ≥0.70 as previously described by O’Connor, et al. All statistical procedures were performed using SPSS 20.0 software (Chicago, IL) and an alpha probability level of <0.05 was adopted throughout. In addition, for all statistical analyses not meeting the sphericity assumption for the within-participant analyses, a Huynh–Feldt correction factor was applied to the degrees of freedom to adjust (increase) the critical *F* value to a level that would prevent the likelihood of committing a type I error.

## Results

### Participants

At baseline, there were no significant differences between groups with regards to height (cm) (CrM-CHO: 177.3 ± 7.4 vs. CHO: 175.1 ± 5.0, *p* = 0.451), body weight (kg) (CrM-CHO: 88.2 ± 12.3 vs. CHO: 94.4 ± 15.3, *p* = 0.332), and age (years) (CrM-CHO: 61.4 ± 5.0 vs. CHO: 60.7 ± 5.4, *p* = 0.762). A Shapiro–Wilk test was used to test for the normality of data and given non-significance was found; we were able to reject the alternative hypothesis and conclude that the data came from a normal distribution.

### Nutritional intake analysis

All participants were instructed to consume their usual diet during the 12-week course of the study. Dietary analysis does not include information related to the supplement ingested. However, monitoring of supplement logs and verbal confirmation of supplement compliance (data not shown) indicated that supplementation compliance was 100 %. Independent sample* t* tests were utilized to analyze all relevant dietary variables prior to commencement of supplementation and the resistance training program. Table 1 illustrates that at baseline (T1), there were no significant differences observed between groups for total daily caloric intake, macronutrient intake of protein, or carbohydrate. Following supplementation, a multivariate analysis revealed a strong trend towards significance for time [Wilk’s Lambda = 0.057, *F*(4,12) = 5.514, *p* = 0.056, effect size (*η* ^2^) = 0.943, power = 0.632]. Univariate analysis detected a significant time effect for dietary fat intake (*p* = 0.025, *η* ^2^ = 0.189, power = 0.727), with subsequent post-hoc analysis revealing significant increases in dietary fat intake from T2 to T4 (*p* = 0.009) and T3–T4 (*p* = 0.013). In addition, dietary fat intake was significantly higher in the CHO group compared to the CrM-CHO group at T4 (*p* = 0.04). These results indicate that while both groups increased their dietary fat intake from T2 to T4, the CHO group was ingesting significantly more dietary fat compared to the CrM-CHO group at T4. No other significant main effects for time, group, or group by time interactions were detected for dietary protein and carbohydrate intake.

**Table 1:** Comparison of nutritional intake variables between CrM-CHO and CHO Groups

Variable	Time	CrM-CHO	CHO	Significance
Energy intake (kcal days^−1^)	T1	2,127 ± 423	1,889 ± 374	Group = 0.883
	T2	1,834 ± 681	2,006 ± 600	Time = 0.142
	T3	1,939 ± 454	1,834 ± 528	*G* × *T* = 0.167
	T4	2,024 ± 490	2,432 ± 680	
CHO intake (g days^−1^)	T1	232 ± 61	208 ± 60	Group = 0.901
	T2	223 ± 120	241 ± 119	Time = 0.885
	T3	237 ± 53	211 ± 97	*G* × *T* = 0.603
	T4	228 ± 83	241 ± 139	
Protein intake (g days^−1^)	T1	98 ± 34	83 ± 18	Group = 0.829
	T2	84 ± 28	78 ± 8	Time = 0.161
	T3	75 ± 15	73 ± 13	*G* × *T* = 0.449
	T4	91 ± 19	108 ± 74	
Fat intake (g days^−1^)	T1	95 ± 36	81 ± 24	Group = 0.773
	T2	68 ± 25	84 ± 29	Time = 0.056
	T3	76 ± 31	80 ± 25	*G* × *T* = 0.207
	T4	83 ± 28	116 ± 32^†,‡,§^	

Data are presented as mean ± standard deviation

^†^ Significantly different from T2 (*p* < 0.05)

^‡^ Significantly different from T3 (*p* < 0.05)

^§^ Significantly different between groups (*p* < 0.05)

### Total lifting volume analysis

All participants were instructed to record, for each exercise, the number of repetitions and weight lifted at each training session. Total lifting volume for each group reflects the total number of repetitions multiplied by the total weight lifted performed by each participant for each exercise (4 weeks: CrM-CHO: 118,780 ± 25,485 kg, CHO: 129,500 ± 33,137 kgs, *p* = 0.500; 8 weeks: CrM-CHO: 130,060 ± 38,313 kgs, CHO: 147,000 ± 40,384 kgs, *p* = 0.422; 12 weeks: CrM-CHO: 142,220 ± 55,236 kgs, CHO: 156,630 ± 41,715 kgs, *p* = 0.331). Independent sample *t* tests were utilized to analyze the total lifting volume at baseline, and repeated measures ANOVA was utilized to assess changes in total lifting volume over time. A statistical trend for time [*F*(2,12) = 3.414, *p* = 0.067, effect size(*η* ^2^) = 0.363] was detected indicating both groups increased their training output following 12 weeks of resistance training.

### Muscle strength analysis

**Fig. 1:**
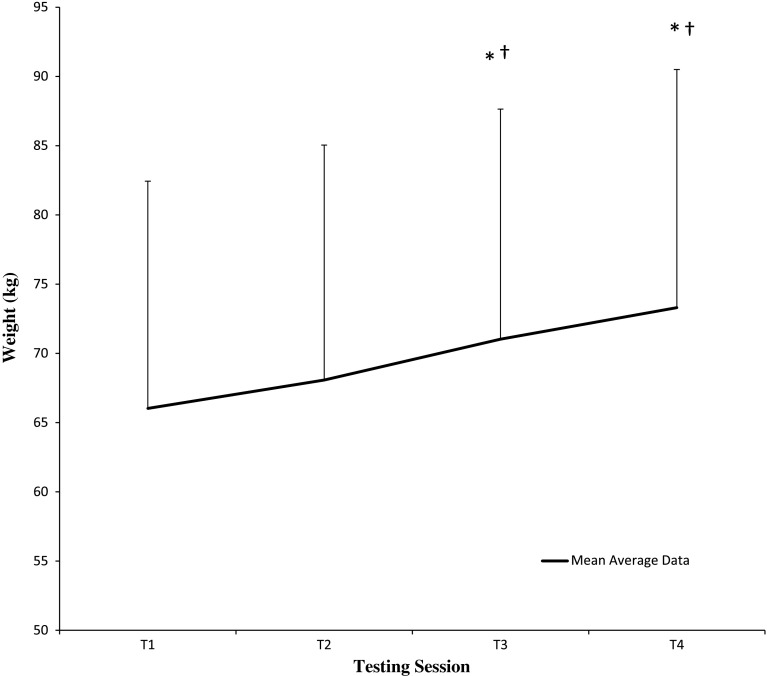
Time course of 1RM bench press. Data (mean ± SD) represents 1RM bench press for both CrM-CHO and CHO groups, including the mean average for both groups combined, following 12 weeks of resistance training. *Asterisk* significantly different from baseline (T1) (*p* < 0.05). *Dagger symbol* significantly different from T2 (*p* < 0.05). Please note that *symbols* used are a reflection of pairwise analysis for time effects and thus differences between mean averages for both groups combined (mean average *line* in figure) at each time point

**Fig. 2:**
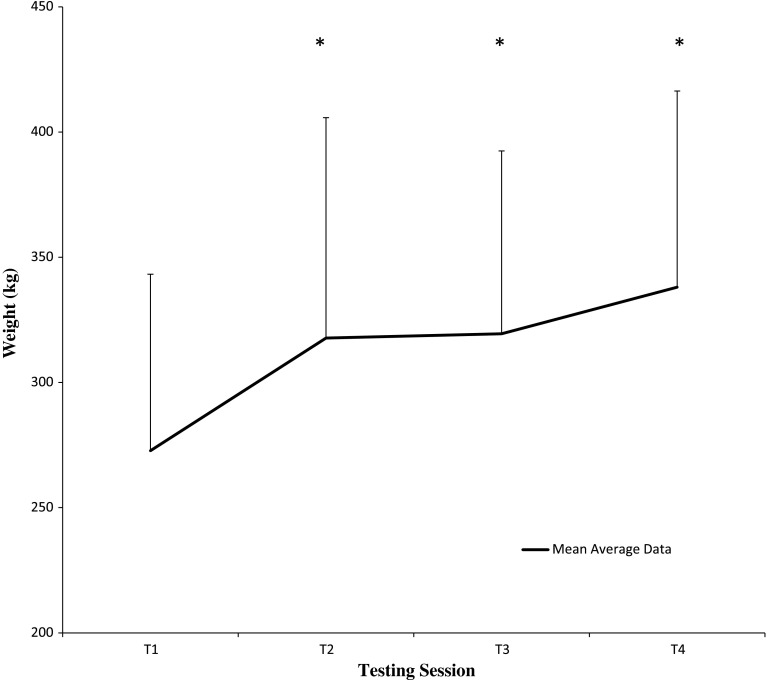
Time course of 1RM leg press. Data (mean ± SD) represents 1RM leg press for both CrM-CHO and CHO groups, including the mean average for both groups combined, following 12 weeks of resistance training. *Asterisk* significantly different from baseline (T1) (*p* < 0.05). Please note that *symbols* used are a reflection of pairwise analysis for time effects and thus differences between mean averages for both groups combined (mean average *line* in figure) at each time point

Data for 1RM bench press and leg press are represented in Figs. 1 and 2. Mulitivariate analysis indicated a significant time effect [Wilk’s lambda = 0.289, *F*(6,13) = 5.336, *p* = 0.006, *η* ^2^ = 0.71, power = 0.941] with no significant group or group × time interactions. Univariate analysis revealed a significant time effect for 1RM bench press [*F*(3,16) = 0.016, *p* = 0.016, effect size (*η* ^2^) = 0.468], with subsequent pairwise analysis displaying significant increases from baseline (T1) to T3 (*p* = 0.022), and T4 (*p* = 0.003), and T2–T3 (*p* = 0.022), and T4 (*p* = 0.002). Similarly, univariate analysis revealed a significant time effect for 1RM leg press [*F*(3,16) = 10.543, *p* = 0.012, effect size (*η* ^2^) = 0.664], with subsequent pairwise analysis revealing a significant increase from baseline (T1) to T2 (*p* < 0.05), T3 (*p* < 0.01) and T4 (*p* < 0.05).

### Body composition analysis

Data for body mass (kg), body fat (%), fat-free mass (kg) and fat mass (kg) are presented in Table 2. Multivariate analysis revealed a significant effect for time (Wilks’s Lambda = 0.046, F(4,15) = 12.108, *p* = 0.001, *η* ^2^ = 0.954, power = 0.998) and a trend towards significance for group effect (*p* = 0.078). No group × time interaction was found. Univariate analysis indicated a significant time effect for body mass [*F*(3,16), *p* = 0.030, *η* ^2^ = 0.419] with subsequent pairwise analysis revealing significant increases in body mass between baseline (T1) and T2 (*p* = 0.008), T3 (*p* = 0.005), and T4 (*p* = 0.003) indicating both CrM-CHO, and CHO significantly increased body mass following 12 weeks of resistance training. A strong statistical trend for group was detected for body fat % (*p* = 0.058), indicating higher body fat % in the CHO group compared to the CrM-CHO group throughout the study period. A significant time effect was detected for fat-free mass (*p* = 0.005, *η* ^2^ = 0.212, power = 0.884) with subsequent pairwise analysis revealing significant increases between baseline (T1) and T2 (*p* = 0.004), T3 (*p* = 0.004), and T4 (*p* = 0.018), indicating both CrM-CHO, and CHO significantly increased fat-free mass following 12 weeks of resistance training. Similar to body fat %, a trend towards significance for group was detected for fat mass (*p* = 0.075), indicating higher body fat % in the CHO group compared to the CrM-CHO group during the study period.

**Table 2:** Body composition for CrM-CHO and CHO groups

Variable	T1	T2	T3	T4	Significance
Body mass (Kg)					Group = 0.093
CrM-CHO	88.2 ± 12.4	89.3 ± 11.9*	89.8 ± 12*	89.9 ± 11.8*	Time = 0.030
CHO	94.4 ± 15.3	94.9 ± 15.1*	94.8 ± 15.3*	95.1 ± 15.5*	*G* × *T* = 0.638
Body fat (%)					Group = 0.058
CrM-CHO	26.7 ± 3.5	26.1 ± 3.4	25.6 ± 2.6	26.5 ± 3.8	Time = 0.232
CHO	29.8 ± 4.3	29.3 ± 3.8	29.6 ± 4.2	29.8 ± 4.9	*G* × *T* = 0.538
Fat mass (Kg)					Group = 0.075
CrM-CHO	21.6 ± 4.8	21.5 ± 4.6	21.3 ± 5.0	21.0 ± 4.3	Time = 0.103
CHO	25.8 ± 5.3	25.6 ± 5.0	26.0 ± 5.9	26.2 ± 6.4	*G* × *T* = 0.289
Fat-free mass (Kg)					Group = 0.516
CrM-CHO	56.6 ± 7.1	58.3 ± 7.2*	59.1 ± 7.4*	58.6 ± 7.9*	Time = 0.005
CHO	58.9 ± 11.2	60.1 ± 11.1*	59.6 ± 10.6*	59.4 ± 10.7*	*G* × *T* = 0.769

Data are presented as mean ± standard deviation

* Significantly different from baseline (T1) (*p* < 0.05)

### Serum analysis

Data for serum IGF-1 and free testosterone are represented in Table 3. Multivariate analysis indicated a trend for time [Wilk’s Lambda = 0.276, *F*(9,10), *p* = 0.056, *η* ^2^ = 0.724, power = 0.703]. No significant time, group or group × time interaction was observed.

**Table 3:** Serum variables for CrM-CHO and CHO groups

Variable	T1	T2	T3	T4	Significance
IGF-1 (pg/ml)					Group = 0.093
CrM-CHO	510 ± 300	424 ± 322	532 ± 310	505 ± 304	Time = 0.261
CHO	280 ± 300	280 ± 230	345 ± 245	283 ± 312	*G* × *T* = 0.638
Testosterone (pg/ml)					Group = 0.516
CrM-CHO	114 ± 94	116 ± 84	109 ± 71	123 ± 89	Time = 0.531
CHO	159 ± 110	158 ± 146	126 ± 69	148 ± 126	*G* × *T* = 0.769

Data are presented as mean ± standard deviation

### Skeletal muscle analysis

Data for total myofibrillar protein concentration and muscle fiber CSA are presented in Table 4. A repeated measures ANOVA revealed a significant time effect for total myofibrillar protein content [*F*(3,15) = 3.509, *p* = 0.042, effect size (*η* ^2^) = 0.412] with no significant group or group × time interactions. Subsequent pairwise analysis showed a significantly higher total myofibrillar protein concentration at T4 compared to baseline (*p* = 0.005). Student’s *t* test revealed a trend (*p* = 0.08) for an increase in size of type II muscle fiber CSA following 12-week resistance training. No significant differences were observed between type I and II CSA or following creatine supplementation.

**Table 4:** Muscle variables for CrM-CHO and CHO groups

Variable	T1	T2	T3	T4	Significance
Fiber CSA (μm^2^ 10^−3^)					
CrM-CHO type I	3.1 ± 0.6			4.8 ± 1.1	
CHO type I	3.8 ± 1.5			4.7 ± 0.9	
CrM-CHO type II	2.9 ± 1.1			4.0 ± 0.6	
CHO type II	3.2 ± 0.6			4.0 ± 0.9	
Total myofibrillar protein content (μg/mg muscle)					Group = 0.316
CrM-CHO	7.9 ± 4.0	10.5 ± 8.7	10.1 ± 1.9	12.0 ± 3.4*	Time = 0.042
CHO	8.5 ± 2.2	8.1 ± 2.6	11.7 ± 7.7	11.1 ± 4.4	*G* × *T* = 0.669

Data are presented as mean ± standard deviation

* Significantly different from baseline (T1) (*p* < 0.05)

## Discussion

The primary purpose of this study was to determine if the consumption of creatine in the hour window post-exercise would stimulate greater training-induced increases in lean muscle mass, muscle fiber CSA and myofibrillar protein content concomitant with upper and lower body muscle strength gains compared to CHO placebo ingestion. In the current study, 12 weeks of resistance training increased body mass, fat-free mass and both upper body and lower body strength. In addition, significantly higher levels of myofibrillar protein concentration and larger muscle fiber CSA, albeit not significant, were observed following the 12-week resistance training program. Despite these improvements, ingesting creatine post-exercise did not enhance the observed resistance training-induced changes in body composition and/or muscle strength.

In young and older populations, greater gains in body and lean muscle mass and muscular strength have been observed following a standard creatine supplementation protocol in conjunction with a resistance training program compared to supplementation or resistance training alone (Becque and Lochman 2000; Bermon et al. 1998; Chrusch et al. 2001; Kreider 2003; Willoughby and Rosene 2001). Participants typically consume 0.3 g kg^−1^ or approximately 20–25 g days^−1^ of CrM for 5–7 days (“loading phase”) and 0.07 g kg^−1^ or approximately 2–5 g days^−1^ of CrM thereafter (“maintenance phase”). Although this protocol has been shown to be effective, others have excluded the loading phase and only used a continuous low dosage (i.e., 5–7.7 g days^−1^) and were able to increase lean muscle mass and muscular performance over placebo-supplemented groups (Brose and Parise 2003; Burke et al. 2000).

In the current study following a standard “loading” protocol, CrM ingestion of ~8.8 g post-exercise was unable to enhance body composition (i.e. gains in fat-free mass, loss of body fat), muscle strength, total myofibrillar protein content and/or individual muscle fiber CSA following a 12-week resistance training program. These observations are in agreement with others who have used a lower dosage (3–5.7 g) before and/or after exercise and failed to show any benefit on exercise-induced adaptations to exercise (Candow et al. 2008; Eijnde et al. 2003). Eijnde et al. (2003) instructed their participants to ingest 3 g of creatine (of the 5 g days^−1^) post-exercise for a period of 24 weeks; where as Candow et al. (2008) ingested approximately 2.85 g of creatine (of the approximate 8 g days^−1^) before and after exercise for a period of 10 weeks. Interestingly, when protein (~24 g days^−1^) was combined with the low creatine dosage, greater gains were seen in lean muscle mass and strength compared to placebo group (Candow et al. 2008). However, given the well established effects of protein/amino acid supplementation on muscle protein stimulation, the observed results are most likely due to protein supplementation and not the creatine per se.

Contrary to our findings, Chilibeck et al. (2004) demonstrated greater increases in elbow flexor muscle thickness and lean mass tissue (males only) when creatine was ingested after a single limb training session twice a week for 6 weeks. It is not readily apparent as to why our hypothesis was not supported. It is clear that the dosage used by Chilibeck et al. (2004) was higher and the duration was shorter compared to the current study (~15.5 vs. ~8.8 g, 6 vs. 12 weeks) and thus could explain why the current study didn’t show any significant benefit from the post-exercise creatine supplementation. However, when you average the dosage intake over the entire week, the difference seems to be minor (~4.5 vs. ~3.8 g days^−1^). We decided to use a lower dosage and longer supplement duration because others (Chrusch et al. 2001; Brose and Parise 2003) have demonstrated that following a typical “loading” protocol and/or continuous low dosage (5 g days^−1^) supplementation period combined with resistance exercise, enhancement of body composition and improvements in muscular performance are typically observed. Although not measured in the study by Chrusch et al. (2001), the level of intramuscular creatine has been shown to influence the performance (Snow and McKenna 1998; Casey and Constantin-Teodosiu 1996); with a positive effect following CrM supplementation normally associated with large increases in intramuscular creatine following supplementation. Thus, we wanted to determine if post-exercise consumption (at a low dosage) following a typical “loading” protocol would be effective.

Therefore, the question is: Does a certain level of intramuscular creatine need to be achieved (i.e., via a typical “loading” protocol and/or continuous ingestion) to stimulate muscle growth or can acute muscle uptake of creatine (i.e., following pre- and/or post-exercise ingestion) have a similar effect? A limitation in our study and Chilibeck et al. (2004) is that total intramuscular CrM concentrations were not measured. Unlike Chilibeck et al. (2004), we performed a standard loading protocol at the beginning and can only speculate that total intramuscular creatine levels were increased and maintained throughout the study. However, even if intramuscular levels were increased, we were unable to demonstrate any significant effects from the post-exercise creatine ingestion. Therefore, when supplementing acutely (i.e. pre- and/or post-exercise) dosage amount (i.e. a higher dosage) could be an important determinant of creatine’s effects within the muscle. Unlike protein supplementation where a series of very well designed studies by Stuart Phillip’s lab (Breen and Phillips 2012) and others (van Loon and Gibala 2011) have determined the threshold for protein dosage and maximal protein synthesis; to date, no similar studies have been performed for creatine supplementation. It could be suggested that a certain amount or “threshold” of creatine is needed to regulate muscle growth when taken acutely. Future work into the timing of creatine supplementation should examine different dosage amounts and its ability to acutely regulate hypertrophic signalling.

It should be noted that the CHO group was ~6 kg heavier compared to the CrM-CHO group at baseline. Although this body mass difference was not deemed significant, it could be argued that this difference may have played a role in adaptive responses to the resistance exercise program. However, total lifting volume data indicated that both groups increased their workload by similar amounts (CrM-CHO ~20 % and CHO ~21 %) and taken together with the lean mass changes; we can argue that the body mass difference at baseline did not significantly influence the results of the study. Another interesting observation was higher fat intakes in the CHO group when compared with the CrM-CHO group. It is not readily apparent as to why the CHO group consumed higher amounts of fat. Regardless, there is little evidence to suggest that higher fat intakes would have a significant effect on the adaptations to resistance training. More importantly, no significant differences were shown in total protein intake and total calories, both known to influence muscle growth and adaptations to exercise.

To our knowledge, the current study is the first to examine long-term effects of CrM ingestion on serum anabolic hormones, IGF-1 and testosterone responses to resistance exercise in middle to older men. It is evident from previous literature that both hormones are important to maximize functional adaptations to resistance training (Schroeder et al. 2013). In fact, exogenous administration of testosterone to hypogonadal males has resulted in significant increases in muscle mass and decreases in fat mass as well as increases in muscle strength (Bhasin and Woodhouse 2001). In the current study, baseline serum testosterone and IGF-1 levels were close or within the normal ranges for men: 29.9–269.2 pg/ml, testosterone and 180–810 pg/ml, IGF-1 (Vermeulen 1996). Following the 12-week training and supplementation protocol, no significant changes in IGF-1 or testosterone levels were observed over the course of the study. However, there was a trend towards significance for higher serum IGF-1 between 4 and 8 weeks for both groups (*p* = 0.066). The results of this study are in agreement with previous findings using resistance training only in older men (Nicklas and Ryan 1995; Brose and Parise 2003). Nicklas and colleagues (Nicklas and Ryan 1995) demonstrated that 16 weeks of progressive resistance training in males older than 55 years of age had no significant influence on baseline serum IGF-1, testosterone and growth hormone concentrations. Though serum growth hormone was increased following an acute bout of resistance exercise, serum concentrations of testosterone and IGF-1 were not affected by the resistance training program. Likewise, Izquierdo and colleagues (Izquierdo and Hakkinen 2001) showed no significant changes in serum-free testosterone following a 16-week resistance training program in middle and older aged males. Interestingly, similar to the current study, upper and lower body strength was increased over time. It could be suggested that since blood samples were obtained 48 h following the last bout of exercise, it is likely that serum IGF-1 and testosterone concentrations returned to baseline levels. Indeed, previous studies examining the short-term response to serum IGF-1 and testosterone concentrations following an acute bout of resistance exercise have demonstrated an increase shortly within the recovery period (12–48 h) (Florini and Ewton 1996), with levels normally returning to baseline within 48 h. Furthermore, creatine supplementation and/or resistance exercise are potentially increasing intramuscular IGF-1 and thus enhancing the anabolic status of the cell (Singh et al. 1999; Burke et al. 2008). With this in mind, given the significant increases in lean muscle mass, total myofribillar protein content and upper and lower body strength in the current study; it could be speculated that transient increases in serum and muscle IGF-1 and/or testosterone shortly following the resistance exercise session are eliciting their anabolic effects within the muscle.

In summary, the results of the present study indicate that post-exercise supplementation with CrM combined with a 12-week high intensity resistance training program does not provide greater enhancement of body composition or muscle strength compared to CHO ingestion alone in middle to older males. However, it is clear that high intensity resistance training can significantly enhance body composition and muscular strength as evident by changes at the whole muscle and cellular level, and thus be an effective method at combating muscle wasting (sarcopenia) and frailty, commonly seen in the elderly. Future studies should examine the acute and chronic effects of different dosages of creatine ingestion before and/or after resistance exercise on pathways involved in skeletal muscle growth.

## Abbreviations

1RM: 1 repetition maximum
ACSM: American College of Sports Medicine
CHO: Carbohydrate
CrM: Creatine monohydrate
CSA: Cross sectional area
DEXA: Dual energy X-ray absorptiometry
ELISA: Enzyme-linked immunoabsorbent assay
GH: Growth hormone
ICC: Intraclass correlation coefficient
IGF-1: Insulin-like growth factor 1
MANOVA: Multivariate analysis of variance
RNA: Ribonucleic acid
SD: Standard deviation

## References

[CR1] Antonio J, Ciccone V (2013). The effects of pre versus post workout supplementation of creatine monohydrate on body composition and strength. J Int Soc Sports Nutr.

[CR2] Bales C, Ritchie C (2002). Sarcopenia, weight loss, and nutritional frailty in the elderly. Annu Rev Nutri.

[CR3] Becque M, Lochman J (2000). Effects of oral creatine supplementation on muscular strength and body composition. Med Sci Sports Exerc.

[CR4] Bemben M, Witten M, Carter J, Eliot K, Knehans A, Bemben D (2010). The effects of supplementation with creatine and protein on muscle strength following a traditional resistance training program in middle-aged and older men. J Nutr Health Aging.

[CR5] Bermon S, Venembre P, Sachet C, Valour S, Dolisi C (1998). Effects of creatine monohydrate ingestion in sedentary and weight-trained older adults. Acta Physiol Scand.

[CR6] Bhasin S, Woodhouse L (2001). Proof of the effect of testosterone on skeletal muscle. J Endocrinol.

[CR7] Borst S (2004). Interventions for sarcopenia and muscle weakness in older people. Age Ageing.

[CR8] Bradford M (1976). A rapid and sensitive method for the quantification of microgram quantities of protein utilizing the principle of protein-dye binding. Anal Biochem.

[CR9] Breen L, Phillips SM (2012). Nutrient interaction for optimal protein anabolism in resistance exercise. Curr Opin Clin Nutr Metab Care.

[CR10] Brose A, Parise G (2003). Creatine supplementation enhances isometric strength and body composition improvements following strength exercise training in older adults. J Gerontol Biol Sci Med Sci.

[CR11] Buford TW, Anton SD, Judge AR, Marzetti E, Wohlgemuth SE, Carter CS, Leeuwenburgh C, Pahor M, Manini TM (2010). Models of accelerated sarcopenia: critical pieces for solving the puzzle of age-related muscle atrophy. Ageing Res Rev.

[CR12] Burke DG, Silver S, Holt LE, Smith Palmer T, Culligan CJ, Chilibeck PD (2000). The effect of continuous low dose creatine supplementation on force, power, and total work. Int J Sport Nutr Exerc Metab.

[CR13] Burke DG, Candow DG, Chilibeck PD, MacNeil LG, Roy BD, Tarnopolsky MA, Ziegenfuss T (2008). Effect of creatine supplementation and resistance-exercise training on muscle insulin-like growth factor in young adults. Int J Sport Nutr Exerc Metab.

[CR14] Campbell WW, Leidy HJ (2007). Dietary protein and resistance training effects on muscle and body composition in older persons. J Am Coll Nutr.

[CR15] Candow D, Chilibeck P (2007). Effect of creatine supplementation during resistance training on muscle accretion in the elderly. J Nutr Health Aging.

[CR16] Candow DG, Chilibeck PD (2008). Timing of creatine or protein supplementation and resistance training in the elderly. Appl Physiol Nutr Metab (Physiologie appliquee, nutrition et metabolisme).

[CR17] Candow DG, Little JP, Chilibeck PD, Abeysekara S, Zello GA, Kazachkov M, Cornish SM, Yu PH (2008). Low-dose creatine combined with protein during resistance training in older men. Med Sci Sports Exerc.

[CR18] Casey A, Constantin-Teodosiu D (1996). Creatine ingestion favorably affects performance and muscle metabolism during maximal exercise in humans. Am J Physiol Endocrinol Metab.

[CR19] Chilibeck PD, Stride D, Farthing JP, Burke DG (2004). Effect of creatine ingestion after exercise on muscle thickness in males and females. Med Sci Sports Exerc.

[CR20] Chrusch M, Chilibeck P, Chad K, Davison K, Burke D (2001). Creatine supplementation combined with resistance training in older men. Med Sci Sports.

[CR21] Cooke MB, La Bounty P, Buford T, Shelmadine B, Redd L, Hudson G, Willoughby DS (2011). Ingestion of 10 grams of whey protein prior to a single bout of resistance exercise does not augment Akt/mTOR pathway signaling compared to carbohydrate. J Int Soc Sports Nutr.

[CR22] Cribb P, Hayes A (2006). Effects of supplement timing and resistance exercise on skeletal muscle hypertrophy. Med Sci Sports Exerc.

[CR23] Doherty T (2003). Invited review: aging and sarcopenia. J Appl Physiol.

[CR24] Eijnde BO, Van Leemputte M, Goris M, Labarque V, Taes Y, Verbessem P, Vanhees L, Ramaekers M, Vanden Eynde B, Van Schuylenbergh R, Dom R, Richter EA, Hespel P (2003). Effects of creatine supplementation and exercise training on fitness in men 55–75 years old. J Appl Physiol (1985).

[CR25] Elliot K, Knehans A, Bemben D, Witten M, Carter J, Bemben M (2008). The effects of creatine and whey protein supplementation on body composition in men aged 48–72 years during resistance training. J Nutr Health Aging.

[CR26] Florini J, Ewton D (1996). Growth hormone and insulin-like growth factor system in myogenesis. Endocr Rev.

[CR27] Gotshalk L, Kraemer W, Mendonca M, Vingren J, Kenny A, Spiering B, Hartfield D, Fragala MV, Volek JS (2007). Creatine supplementation improves muscular performance in older women. Eur J Appl Physiol.

[CR28] Hunter G, MCarthy J, Bamman M (2004). Effects of resistance training on older adults. Sports Med.

[CR29] Izquierdo M, Hakkinen K (2001). Effects of strength training on muscle power and serum hormones in middle-aged and older men. J Appl Physiol.

[CR30] Jager R, Harris RC, Purpura M, Francaux M (2007). Comparison of new forms of creatine in raising plasma creatine levels. J Int Soc Sports Nutr.

[CR31] Janssen I, Heymsfield S, Ross R (2002). Low relative skeletal muscle mass (sarcopenia) in older persons is associated with functional impairment and physical ability. J Am Geriatr Soc.

[CR32] Kreider R (2003). Effects of creatine supplementation on performance training adaptations. Mol Cell Biochem.

[CR33] Nicklas B, Ryan A (1995). Testosterone, growth hormone and IGF-1 responses to acute and chronic resistive exercise in men aged 55–70. Int J Sports Med.

[CR34] Olsen S, Aagaard P, Kadi F, Tufekovic G, Verney J, Olesen J, Suetta C, Kjaer M (2006). Creatine supplementation augments the increase in satellite cell and Myonuclei number in human skeletal muscle induced by strength training. J Physiol.

[CR35] Rawson E, Volek J (2003). Effects of creatine supplementation and resistance training on muscle strength and weightlifting performance. J Strength Cond Res/Natl Strength Cond Assoc.

[CR36] Rawson E, Wehnert ML, Clarkson PM (1999). Effects of 30 days of creatine ingestion in older men. Eur J Appl Physiol Occup Physiol.

[CR37] Schroeder ET, Villanueva M, West DD, Phillips SM (2013). Are acute post-resistance exercise increases in testosterone, growth hormone, and IGF-1 necessary to stimulate skeletal muscle anabolism and hypertrophy?. Med Sci Sports Exerc.

[CR38] Singh MA, Ding W, Manfredi TJ, Solares GS, O’Neill EF, Clements KM, Ryan ND, Kehayias JJ, Fielding RA, Evans WJ (1999). Insulin-like growth factor I in skeletal muscle after weight-lifting exercise in frail elders. Am J Physiol.

[CR39] Snow R, McKenna M (1998). Effect of creatine supplementation on sprint exercise performance and muscle metabolism. J Appl Physiol.

[CR40] Stark M, Lukaszuk J, Prawitz A, Salacinski A (2012). Protein timing and its effects on muscular hypertrophy and strength in individuals engaged in weight-training. J Int Soc Sports Nutr.

[CR41] van Loon LJ, Gibala MJ (2011). Dietary protein to support muscle hypertrophy. Nestle Nutr Inst Workshop Ser.

[CR42] Vermeulen A (1996). Decreased androgen levels and obesity in men. Ann Med.

[CR43] Williams M, Branch J (1998). Creatine supplementation and exercise performance: an update. J Am Coll Nutr.

[CR44] Willoughby D, Rosene J (2001). Effects of oral creatine and resistance training on myosin heavy chain expression. Med Sci Sports Exerc.

